# Physician perceptions of recruitment and retention factors in an area with a regional medical campus

**Published:** 2018-03-27

**Authors:** Mylene Levesque, Sharon Hatcher, Denis Savard, Reine Victoire Kamyap, Pauline Jean, Catherine Larouche

**Affiliations:** 1 Centre intégré universitaire de santé et de services sociaux du Saguenay-Lac-Saint-Jean, Québec, Canada; 2 Université de Sherbrooke, Québec, Canada; 3 Université du Québec à Chicoutimi, Québec, Canada; 4 Université Laval, Québec, Canada

## Abstract

**Background:**

The factors that influence physicians to establish and maintain their practice in a region are variable. The presence of a regional medical campus (RMC) could influence physicians’ choice. The objective of this study was to explore the factors influencing physician recruitment and retention, and in particular the role of a RMC, in a region of Quebec.

**Methods:**

A literature review of factors influencing physicians to stay in a rural area was conducted in order to create an interview guide. Questions were divided into sections: general information, family situation, medical training, career choice, current practice, intent to stay in the region, and impact of the RMC. Thirteen semi-structured individual interviews were conducted with practicing physicians. Data were analyzed using QDAMiner.

**Results:**

Recruitment factors were divided into six major themes: type of practice, spousal interest, opportunity for teaching, training in a region, workforce planning, and quality of life. Participants identified positive and negative factors associated with retention. In both cases, family and quality of work environment were mentioned. The RMC was perceived as having important impacts on the quality of professional life, research, medical practice, and regional development.

**Conclusion:**

This study highlights the role of RMCs in physician recruitment and retention via multiple impacts on the quality of practice of physicians working in the same area.

## Abstract (fr)

**Contexte:**

Les facteurs influençant les médecins à s’établir et à rester dans une région sont variables. La présence d’un campus médical régional (CMR) pourrait influencer ce choix. L’objectif de cette étude était d’explorer les facteurs de recrutement et de rétention influençant les médecins ayant choisi de pratiquer dans la région du Saguenay-Lac-Saint-Jean au Québec, en particulier le rôle du CMR.

**Méthodes:**

Une synthèse de la littérature a permis d’identifier différents facteurs influençant les médecins dans leur choix de lieu de pratique. Un guide d’entrevue a été élaboré à partir de ces facteurs. Les questions étaient séparées selon les sections suivantes: informations générales, situation familiale, études médicales, choix de carrière, pratique actuelle, intention de rester dans la région, impact du CMR. Treize entrevues semi-dirigées individuelles ont été réalisées avec des médecins en pratique. Les données ont été analysées avec QDA Miner.

**Résultats:**

Les facteurs influençant le recrutement étaient séparés en six thèmes majeurs : type de pratique, intérêt du conjoint, opportunité d’enseigner, formation en région, planification gouvernementale des effectifs médicaux et qualité de vie. Les participants ont identifié des facteurs de rétention négatifs et positifs. Ceux-ci concernaient la famille et la qualité de l’environnement de travail. D’après les participants, le CMR avait un impact direct sur la qualité de la vie professionnelle, la recherche, la pratique médicale et le développement régional.

**Conclusion:**

Cette étude a permis de mettre en évidence le rôle des CMRs dans le recrutement et la rétention via de multiples impacts sur la qualité de pratique des médecins exerçant dans la même région.

## Introduction

There is a worldwide shortage of physicians in rural areas.^1^ In Canada, less than 8% of physicians practice in rural regions, whereas 19% of Canadians live in those areas.^2^ In 2015, 9.2% of physicians from the province of Quebec were practicing in areas outside a metropolitan centre, whereas 19% of the population live in those areas.^3,4^ According to the World Health Organization (WHO), “all citizens should have an equal opportunity to be healthy.”^1^ The discrepancy between population distribution and physician distribution can lead to health inequality.

Over the years, different strategies have been used to encourage physicians to establish their practice in outlying areas. Financial incentives and adaptation of medical student selection criteria are amongst the strategies that have been used.^5^ Another strategy has been the establishment of regional medical campuses (RMCs).^6^ This development in medical education is supported in part because several studies have found that rural experiences during medical school positively influenced future rural medical practice.^7-9^ Some authors questioned medical students on their intentions to practice in rural areas, and found a positive impact of regional education. Myhre et al. found that students were more likely to intend to practice in a regional or rural community after their regional rotation.^10^ Isaac et al. found that two or more years in a rural medical school were associated with intentions to practice in a rural location.^11^ Other authors found that students who studied in or were exposed to a rural area were more likely to actually practice in rural locations.^12-14^ Brokaw et al. found that training in a two-year basic science RMC was significantly predictive of practicing outside a metropolitan area.^15^ These studies point to the potential importance of a RMC in the choice of its graduates’ practice location.

In 2006, the University of Sherbrooke established two RMCs, one in the province of Quebec, and one in the province of New Brunswick. The RMC in Quebec was established in the Saguenay-Lac-Saint-Jean (SLSJ) region. The SLSJ region is not qualified as entirely rural as its population is 275 000.^16,17^ Nevertheless, the region has a vast area of 95 892 km^2^, where population is spread in 49 municipalities, 10 non-organized territories, and two First Nations communities.^18^ The RMC is located in the municipality of Saguenay, which has a population of 150 949.^16^ The campus is located 430 kilometers from the main campus in Sherbrooke, and 200 kilometers from Quebec City, the nearest metropolitan area. The RMC houses a fully distributed medical program, the Saguenay Medical Education Program, which partners with the regional university and the main teaching hospital so that students can complete their four years of medical education at this campus.

In the ten years since the beginning of the program, hospital and provincial data have shown that the chronic shortage of family physicians and specialists in the SLSJ region has been largely overcome, meeting workforce targets.^19^ However, this phenomenon cannot be related to the recruitment of graduates from the RMC, who entered into practice only after 2012 (those in family practice) or after 2015 (those in other specialties). Two research questions emerged for the local research team: 1) What are the perceived recruitment and retention factors for physicians who settled in the SLSJ region over that first ten-year period of the RMC? 2) What has been the influence of the presence of the RMC on physician choice of practice location?

Thus, the aim of this study was to explore physician perceptions of the factors influencing recruitment and retention, including the role of the RMC. We did not seek to specifically question physicians that were trained at a RMC on the role of the RMC on their choice of practice. Instead, we asked physicians what effect the existence of a RMC had on their decision to come and stay in the region. We did not find other studies looking specifically and intentionally at the question of recruitment and retention in this context.

## Methods

### Study design

An exploratory qualitative methodology with semi-structured individual interviews was chosen to explore the perceptions of physicians recruited between 2006 and 2016 in the SLSJ region. Focus groups were not carried out for feasibility reasons, given complicated scheduling. After discussion with the ethics committee of the Université du Québec à Chicoutimi (UQAC), the project did not require ethical approval as it was considered in the domain of program evaluation, and an exemption certificate was obtained from the committee. Nonetheless, consent forms were still distributed to and completed by participants. Subsequently, approval from the ethics committee was asked for and obtained for secondary use of the data in view of the publication of this article (file number 602.409.05).

### Participants

Participants were recruited on a voluntary basis by email, phone or via snowball effect, forming a non-probabilistic sample. A list of physicians recruited in the SLSJ region between 2006 and 2016 was obtained via the regional health authority administrative database. A list of potential participants corresponding to the variables desired for the sample was drawn up. Initial invitations were sent out by email (contact information was available via the hospital list serve). When there was no response, contact was made by phone via the hospital operator. Participants were selected based on various characteristics for maximum variation: the sample was to be comprised of family physicians and specialists; men and women; teaching and non-teaching physicians; physicians originating from the SLSJ, from other regions, and from metropolitan areas; and finally, physicians trained at the University of Sherbrooke and at other universities.

### Study protocol

The interview guide was based on a literature review of studies on recruitment or retention factors of physicians practicing in semi-rural, rural, or remote areas. Inclusion criteria for the articles were the following: studies describing or measuring recruitment or retention factors, or both. Articles were excluded if they were not written in English or French, were written before 2006, or came from a significantly different medical education system. Fourteen articles were used to construct a conceptual framework (Table 1). Based on this framework, the interview guide explored the following themes: general information, family situation, medical training, choice of career location, recruitment factors, current medical practice, intent to stay in the SLSJ region, and the impact of the RMC. Interviews were planned to last about 60 minutes. Two researchers conducted the individual interviews in French between June and August 2016.

**Table 1:** Conceptual framework for the interview guide

Factors	Details
Personal	Rural origins Motivation, interest, intentions Lifestyle
Family	Proximity of family Employment of spouse
Professional	Medical training in a region Workload Support for practice Type of practice
Community	Living environment (access, leisure, support)
Economic	Recruitment incentives
Recruitment process	Selection process Professional experience References by colleagues
Workforce planning	Incentive program Quotas Obligations

### Data analysis

Interviews were conducted until data saturation. The interviews were audio-recorded, transcribed, and analyzed using a thematic analysis approach.^20^ As our interview guide was based on a theoretical framework developed by our team, and based on a literature review, the coding process was deductive, but considering that we were receptive to the emergence of new themes, the process was also inductive. The data were analyzed by both interviewers. Initial coding of the data set was done separately and then researchers met, compared and contrasted their findings in order to reach a team consensus on themes forming a framework applied to all interviews. The data were processed using the QDA Miner qualitative analysis software. Verbatim extracts were selected and then translated into English by members of the research team.

## Results

### Participants

Thirteen physicians participated in the study: five were women, and eight were men. Interviews lasted from 35 to 90 minutes with a mean duration of 41 minutes. The mean age of participants was 35.4 ±6.4 years. Three participants were family physicians and ten were specialists. Nine participants were involved in teaching in the Saguenay Medical Education Program, two were involved in clinical training, and two were not involved in teaching at all. Four participants were from the SLSJ region, one from Quebec City, one from Montreal, eight from other regions of Quebec, and three from other countries. Table 2 summarizes participants’ educational background. Two participants had completed their undergraduate medical education at the Saguenay RMC. Nine participants had family or extended family members in the SLSJ region. All participants were practicing in the Saguenay part of the region where the main teaching hospital and the RMC are located.

**Table 2:** Participant educational background

	SLSJ region	Other regions of Quebec	Metropolitan areas*	Other countries
**Primary and secondary schooling**	6	2	2	3
**Undergraduate medical education**	2	2	7	2
**Postgraduate medical education**	1	6	4	2

* Metropolitan areas: Montreal and Quebec City, as defined by the provincial government municipal organization charts.^17^

### Study findings

#### Recruitment factors

Thematic analysis of participant interviews permitted the identification of six major themes associated with recruitment factors: *type of practice, spousal interest, opportunity for teaching, training in a region, workforce planning*, and *quality of life*.

##### Type of practice

A majority of participants stated that they had chosen the region for the type of practice found in the SLSJ. For some of them, it was the major decisional factor. Participants qualified the practice in the region as collegial, well organized, and a good work environment. They also indicated that the practice was stimulating, large in scope, and team-based. They found that cases were more diversified and more complex, that the practice was broader, more humane, and patient-centered. Also, some mentioned that the workload was not too heavy. All participants confirmed their access to the necessary material and resources for their practice; in other words, they did not feel they had fewer resources than in other hospitals. Some found that their access to technical platforms, like operating rooms or ultrasound machines, was less restricted than in larger centers. Physicians mentioned that they did not frequently need to refer their patients to health care centers outside of the region, but, for some participants, access to specialists was more difficult. One participant said: “Yes, we are perhaps more often left to ourselves and sometimes it is a little more stressful because we have no specialist support, but the rest, the collegiality and mutual assistance that we have between us, the intensivist will go down, come and find us, will help us even if it is not his specialty, even if … they will not hesitate to help us to save a patient.” (p12)

##### Spousal interest

Several participants talked about the importance of their spouse in their decision to practice in the region, and it was the major factor for some of them. Spousal employment, opportunity to study, or attractiveness of the region were stated as important recruitment factors for participant spouses. One participant said that possible employment for his spouse was a major factor in their decision to come to the SLSJ region: “So she also had to find a place to work; I think it is a major factor that the spouse can find a job. When we visited the hospital, there was an opportunity to hire a nurse practitioner in a clinic. We felt that the job opportunity was good for both of us, and we decided to go for it.” (p3)

##### Opportunity for teaching

Several participants expressed that the possibility of teaching in the RMC was an important motivator in choosing the SLSJ region. This was also the determining factor for some. One participant stated: “Well, I was sure that it was something that really mattered to me, teaching. I find it very important. I think that′s what trains the doctors of tomorrow and that′s what will motivate people.” (p1) Participants declared that teaching permitted them to keep their knowledge up to date, that it was stimulating, and that it gave meaning to their practice.

##### Training in a region

Several participants explained that experiencing one or more rotations in the SLSJ region during their medical training led them to discover life and medical practice in the region. As one participant put it: “[…] we can keep a doctor here because he has come to do his M.D. here; he has loved the area so much that even though he is not interested in medical teaching, he is interested by the medical practice in the region." (p9)

##### Workforce planning

Some participants identified the role of workforce planning by government as a recruitment factor. One participant was obligated to practice in the region, and one came because of the availability of positions. One physician told us: “I found on the Internet: ‘Recrutement Santé Québec.’ I thought, there is a possibility of working in my field; I sent a resume, and they answered me … yes. So that′s how … it was a little accidental … The only place we could possibly accept you is in Saguenay. So, it was like that. I did not really have much choice.” (p2)

##### Quality of life

Some participants considered quality of life as an important factor in their recruitment. However, it was not a determining factor for participants. They noted that the region was good for outdoor activities, access to services (health, education, entertainment), and had a good overall quality of life. One participant stated: “Well I think it′s the best quality of life that we can have. We do not have the stress of the city, but at the same time the town is not too small, so we still have accommodations. I think it′s really a quality of life that is perfect; we do not have traffic, we have outdoor activities, we still have a bit of culture.” (p12)

#### Retention factors

Participants were asked about their intent to stay in the region, and what could possibly lead them to leave. Positive and negative factors were therefore identified by participants. There were three major themes associated with the intent to stay in the region, and two themes associated with the possibility of leaving the region.

##### Positive retention factors

The positive factors were related to *family, quality of life*, and *quality of the work environment*. Family-related reasons were associated with the integration of family members in the region, notably the integration of children in their school, and the integration of a spouse in the community. One participant said: “All the stories that I have heard of doctors who left the region, it was because their spouse did not integrate well in the region. […] now that our integration is done, that my wife is integrated, that she is comfortable, that her mother has moved to the region […], now we are constructing our lives, our family begins.” (p2) As illustrated by this quote, the proximity of family was also an important retention factor for participants.

Quality of life was also very important in the retention of physicians: “Of course, our goal would be to stay in the area, because we love our life here, our quality of life and our jobs, for my partner and for me.” (p5)

The quality of the work environment was a major factor of retention for participants. Physicians said they were staying in the region because they felt commitment to the RMC, and they had the opportunity to teach. They also mentioned that there were interesting career possibilities in the region: “My career perspective here is very interesting, and I have alternatives, opportunities to change my career, either in research, teaching, or in the clinical setting.” (p10)

##### Negative retention factors

Participants were asked about the possible reasons that could lead them to leave the region. Two major factors were identified: *family* and *work environment*. Physicians said they would leave the region if their spouse lost their job or was unable to find one, if they separated from their current partner or found a partner who did not live in the region. One participant said: “In fact, the only factor that would make us leave would be the loss of his job.” (p5)

The second theme involved the work environment. Participants said they would leave the region if there were major changes in practice:

I think what might affect me would be something major in the practice. […] For now, it is difficult to motivate people to practice in family medicine. But it has nothing to do with the program or with my current practice, it is only the current political context that is difficult. […] If I were forced to drop the teaching, to go to a big medical office, to let go half of my emergency practice, well it sure would play a role (in the decision to quit). (p.1)

#### RMC impacts

Participants estimated that the Saguenay RMC had a major impact on physician recruitment and retention in the region. Four major themes emerged from the data analysis. Participants thought that the RMC had an impact on the *quality of professional life, research activities, medical practice in general*, and *regional development*.

##### Impacts on the quality of professional life

The RMC had various impacts on participant satisfaction with their professional life. Participants reported that they liked to teach, transmit their knowledge, and see the students learn. One participant spoke of the importance of teaching for his motivation:

[…]it happens to us, to become a little more pessimistic, bleak with the big reforms, the minister who shakes up the whole system, not necessarily for the best when we see on the ground the perceptions of the population […]. But these students, they arrive, and they are pure, for them it’s the beauty of medicine and knowledge, and helping patients. So, it brings us back to basic values and it keeps us in that mindset. It pushes us to perhaps forget our little political frustrations and come back to the real issues when we teach, which are the contact with the patient, the relationship we have, and the quality of care that we dispense. (p4)

One participant stated how important it was to him to be a role model through his work in the Saguenay program. Some also mentioned that clinical teaching was part of continuing medical education, and being a preceptor was part of their role as physicians.

##### Impacts on research activities

Impact of the RMC on research activities was less significant for participants, but it was mentioned as being potentially important. There were few participants in the study who were involved in research. Most of the participants could speculate on the effect of the RMC on research activities, but had no concrete experience or examples. However, one participant did state that the presence of the RMC had a positive impact on his research project. Others thought that the presence of the RMC facilitated the development of research by increasing the number of trainees participating in research projects:

I think that it helps those who are doing research; I think it structures their teams and the way they work. […] So to have the Faculty (of medicine), it gives manpower, it brings people who have interests and it develops regional research interests, because there are not many research “regions” in Quebec, really, really not. So, I think the Faculty gives resources and some help, maybe a little help for researchers and research teams that might not have it otherwise, that would be more fragile if they were alone in a hospital. (p.10)

##### Impacts for medial practice in general

According to participants, the RMC had increased the retention of students, the number of specialists, and had helped build an academic environment. One participant mentioned that having students and residents increased his motivation to maintain up-to-date knowledge:

I think that when you practice alone in your small office and you never see anyone, you keep yourself less up-to-date because it is less motivating. For me, it would certainly make a difference. When you have students, and you’re teaching at the Faculty, well you must return to your books, read more, prepare your courses, prepare your journal club, because your residents will be aware of the latest scientific article, and you will want to answer their questions correctly. You also do not want to give a lecture to residents and not know what you′re talking about. It′s certain that it motivates you and forces you to stay up-to-date. (p.1)

Other physicians also stated that the RMC had improved the quality of health services, created a dynamic atmosphere in the region’s hospitals, and had made the region more attractive for new physicians.

##### Impacts on regional development

Participants mentioned economic impacts on the region through employment and investments. They also stated the positive impact of the RMC on the health of the population, by increasing access to medical specialties, retaining new physicians, and improving the quality of health care. It was felt that the RMC also improved the prestige of the region. One participant also mentioned the increased accessibility of medical studies: “I think that having a Faculty of medicine offers a ‘service’ to people coming from the region or regions nearby, to be able to study medicine at a lower cost. In the sense that some people who come here, who do not have a lot of money, could say to themselves … sometimes it can play, weigh in the balance between I′m going to go to nursing school or I′m able to afford medical studies.” (p12)

## Discussion

Results of this study highlighted factors associated with physician recruitment and retention in outlying regions such as the SLSJ. Recruitment factors identified by participants were the following: type of practice, spousal interest, opportunity for teaching, training in a region, workforce planning, and quality of life. Factors influencing retention were divided into positive and negative factors. Perceived positive retention factors were related to family, quality of life, and quality of the work environment. Potential negative retention factors were also related to family and the work environment. Finally, for participants, the RMC impacted their choice of practice location and their desire to stay in the region via the quality of professional life, research activities, medical practice in general, and regional development.

Several findings from our study were congruent with the literature. As expected, factors related to spouse and family had a significant influence on the choice to come and to stay in the region. Many authors have reported the same types of results.^21-28^ Cameron et al. found that spousal and family support emerged as significant factors from the four communities that they questioned.^29^ In our study, quality of life was also identified as an important recruitment and retention factor. Preference for a rural lifestyle was mentioned by physicians in many studies.^21,23,24,30,31^ Finally, being exposed to regional practice in training was felt by the participants in our study to have a major impact on physician recruitment. Rural exposure was also an important recruitment factor in the literature.^14,22,30,32^

Our study also highlighted factors that are less often reported in the literature. The type of practice was mentioned by nearly all participants. The ability to have a large scope of practice, autonomy and continuity of care were important recruitment and retention factors. Some authors have previously reported the importance of the type of practice in the decision to come and stay in a region,^21,23,30,33^ but it was not as major as in our interviews, in which almost all participants spoke extensively about this subject. A recent literature review rated the scope of practice as a very low predictor of recruitment.^34^ One possible explanation is that this topic of research is relatively new, and less evidence is available, as only few studies presented results on this factor.^34^ Financial factors were not cited by our participants, but are sometimes considered as recruitment and retention factors in the literature.^35^ In accordance with our findings, MacQueen et al. attributed a very low grade of evidence to salary as a recruitment factor.^34^ On the other hand, Verma et al. rated financial incentives as the strongest factor in recruitment intervention.^36^ Thus, financial incentive does not seem to be a consistent factor of recruitment in the literature. Furthermore, rural upbringing was not evoked as a significant influence in our interviews as opposed to what is seen in the literature.^11,13,15,37-41^

Our study highlighted the role of the RMC in the recruitment and retention of physicians. Many participants mentioned the importance of teaching and for some, it was a major recruitment and retention factor. To our knowledge, this is not a factor that has been previously identified in the literature. The presence of the RMC was seen to have a positive impact on student interest in regional practice, but participants also felt that it played a key role in creating an attractive academic environment for physicians interested in teaching. Our findings also suggest a positive impact of the RMC on medical practice, on quality of care, on the prestige of the region, and, to a lesser extent, on research. One study reported a positive impact of RMCs on clinician job satisfaction and quality of health care.^13^ The same authors highlighted the importance of RMCs in sustainable workforce development in rural areas.

Our study had some limitations. For one, the interviews were conducted with physicians already in practice, so there might be a bias of memory concerning recruitment factors. This phenomenon might be reflected by the fact that recruitment factors were similar to retention factors - participants could have mixed both types of factors as they did not necessarily recall precisely what initially drove them to come to the region. On the other hand, the study sample did contain some relatively new physicians who had not been in practice for a very long time. Moreover, our sample mostly consisted of specialists; it would have added to the validity of our results to have more family physicians. We also only recruited physicians that were practicing in the SLSJ region, and it could have been interesting to question physicians who had left the region. It would also have increased the validity of our findings to include physicians that are practicing in other regions where a RMC has been established. Research on this topic could benefit from further mixed study designs to better understand the impacts of RMCs in regional areas and to increase the generalizability of the findings.

### Conclusion

This study identified different recruitment and retention factors important to physicians practicing in a regional area. It may also call attention to attractive aspects of regional practice that physicians not previously exposed to such practice might not suspect. This study adds to the relatively new and growing body of evidence that the presence of a RMC positively contributes to physician workforce development. Our findings underline the importance of RMCs, not only through the practice location of their graduates, but via their impacts on the work environment and quality of professional life of physicians practicing in the same area. Thus, the results of this study could be useful for decision makers and stakeholders when making critical choices about the most effective strategies to improve physician workforce in underserved regions.
